# Development of Analytical Quality by Design Compliant Chaotropic Chromatography Method for Ziprasidone and Its Five Impurities Determination

**DOI:** 10.3390/ph16091296

**Published:** 2023-09-14

**Authors:** Milena Rmandić, Đorđe Vasilić, Marija Rašević, Mira Zečević, Biljana Otašević, Ana Protić, Anđelija Malenović

**Affiliations:** 1 Department of Drug Analysis, Faculty of Pharmacy, University of Belgrade, Vojvode Stepe 450, 11000 Belgrade, Serbia; milena.rmandic@pharmacy.bg.ac.rs (M.R.); mira.zecevic@pharmacy.bg.ac.rs (M.Z.); biljana.otasevic@pharmacy.bg.ac.rs (B.O.); ana.protic@pharmacy.bg.ac.rs (A.P.); andjelija.malenovic@pharmacy.bg.ac.rs (A.M.); 2 Medicines and Medical Devices Agency of Serbia, Vojvode Stepe 458, 11000 Belgrade, Serbia; djordje.vasilic998@gmail.com

**Keywords:** chaotropic chromatography, ziprasidone, impurities, analytical quality by design, robustness testing of method quantitative performances

## Abstract

In this study, an AQbD-compliant chaotropic chromatography method for ziprasidone and the determination of its five impurities was developed. The influence of critical method parameters (initial and final methanol fraction in the mobile phase, gradient duration) on the set of selected critical method attributes (*t__imp. V_*, *t__imp. V_ − t__imp. I_*, *S* and *<W_USP_>*) was studied by Box–Behnken design. The errors resulting from the calculation of the model coefficients were propagated to the selected responses by Monte Carlo simulations, and their predictive distribution was obtained. The design space was computed (π ≥ 80%), and a working point was selected: initial methanol fraction 38.5%, final methanol fraction 77.5%, and gradient duration 16.25 min. Furthermore, the quantitative robustness of the developed method was tested using the Plackett–Burman design. *P__imp II_* and *P__imp V_* were found to be significantly affected, the first by mobile phase flow rate and the second by gradient duration. Finally, the method was validated, and its reliability for routine quality control in capsules was confirmed.

## 1. Introduction

**Figure 1:**
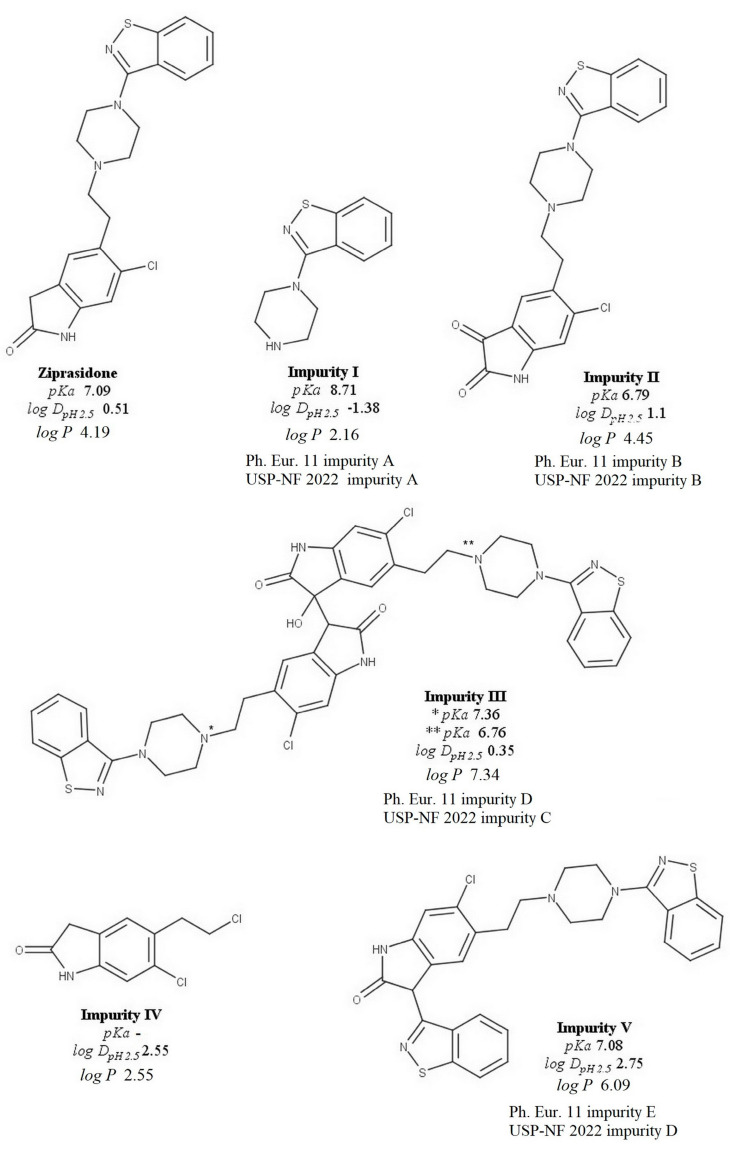
Chemical structures, *logP*, *logD_pH_*_2.5_, and acidic–basic properties of the analyzed solutes.

Ziprasidone (ZPS) is an atypical antipsychotic of the benzisothiazolyl piperazine type that belongs to the second generation of antipsychotics used to treat schizophrenia. Chemically, it is 5-[2-[4-(1,2-benzisothiazol-3-yl)piperazin1-yl]ethyl]-6-chloroindolin-2-one hydrochloride hydrate [1]. Since it is a very water-insoluble compound, it is administered in the form of a salt, usually hydrochloride [2]. It is a weak base (*pKa* 7.09 ± 0.10) with a partition coefficient of *logP* 4.19 and *logD_pH_*_2.5_ 0.51 [3]. The structures of ZPS, its degradation products (impurities II, III, and V), and process-related impurities (impurities I and IV) are shown in Figure 1. In the Ph. Eur. 11 Transparency List [4], five impurities (A–E) of bulk ZPS hydrochloride can be found. In addition, in USP-NF 2022 [5], six impurities (A, B, C, D, F, and the process impurity chlorindolinone) are investigated in raw materials and capsules of ZPS hydrochloride. The relationship between the impurities in Ph. Eur. 11 and USP-NF 2022 is shown in Figure 1.

The analysis of ZPS hydrochloride-related substances is very specific. Due to a significant difference in the impurities’ polarities, two separate chromatographic systems are official in Ph. Eur. 11 [4] for the determination of these analytes in bulk drug. System A is for the analysis of three early eluting polar impurities (A, B, and C), and system B is for late eluting lipophilic impurities (D and E). On the other hand, in USP-NF 2022 [5] for the analysis of impurities in ZPS hydrochloride bulk drug and in capsules, the single one RP-HPLC gradient elution method with a total run time of 75 min is official.

In addition, few other methods are described in the literature that involve the determination of the content of ZPS and its five impurities. Although the developed TLC densitometric method [6] for the simultaneous determination of ZPS and its five impurities is the simplest chromatographic separation technique, its insufficient sensitivity and the use of toluene as mobile phase limit its applicability. A sensitive and reproducible RP-HPLC gradient elution method was developed and validated for the determination of ZPS and its five impurities [7]. However, the separation was performed with a mobile phase consisting of acetonitrile and buffer (aqueous KH_2_PO_4_ solution with triethylamine). The same group developed the highly sensitive UHPLC-MS/MS gradient elution method for the simultaneous quantification of ZPS and its five impurities and with the possibility to characterize the unknown degradation product [8]. Finally, they have also published a comparative study of the performance of the UHPLC-MS /MS and HPLC-UV methods for the analysis of ZPS and its major impurities [9].

The methods available in the literature for the analysis of ZPS and its five impurities have a drawback that is mainly related to the complicated and time-consuming chromatographic analysis [4,5], the lack of sensitivity [6], the use of phosphate salt and trietylamine as mobile phase modifiers [7], which are not recommended because of their influence on the lifetime of the column and chromatographic system, or the use of equipment that is not easily accessible in routine drug quality control [9]. Moreover, the basic properties and significant polarity differences in these solutes pose additional challenges for their RP-HPLC separation and determination. A well-established approach to adjust retention and to improve peak symmetry and separation efficiency of basic solutes in RP-HPLC systems is the addition of column-friendly agents, referred to as chaotropic agents, to the mobile phase [10,11,12,13,14]. Therefore, the aim of this research was to develop a reliable and robust chaotropic chromatography method suitable for routine quality control of ZPS and its five impurities (Figure 1) in capsules.

Contemporary approaches to pharmaceutical regulation propose the use of a risk-based Analytical Quality by Design (AQbD) approach to analytical method development [15]. The new ICH Q14 guideline proposes that method development should be based on monitoring parameters that have an impact on HPLC separation of substances. It clearly suggests that HPLC method development should be based on the AQbD approach, which uses Design of Experiments (DoE) principles [16]. DoE results in fitting data to mathematical models that are closely related to the problem/system under study. This is the basic assumption of the DoE methodology, and generalization, i.e., application of the obtained models to other active pharmaceutical agents and their impurities, is neither possible nor to be expected. Developing a method based on AQbD is important in several ways: the occurrence of out of specification (OOS) results is reduced, making the method more profitable after development and validation because it does not require further modification, and the results obtained with this method have higher validity [17].

The convergence of science and compliance results in a reliable and robust AQbD method that ensures an efficient and scientifically sound analytical control strategy. This approach estimates the effects of input variables or critical method parameters (CMPs) on a set of selected performance criteria or critical method attributes (CMAs) to create the design space (DS). Working within the DS implies that no significant changes in CMAs should be observed as a result of small, intentional changes in CMPs. In this study, we used robust optimization and risk assessment of the method to meet the predefined high probability (π) acceptance criteria. Monte Carlo simulations were performed to propagate the uncertainties to the CMAs and obtain their predictive distribution. A working point was selected from DS, the robustness of the quantitative performance of the developed method was investigated, and, finally, the developed method was validated to demonstrate its suitability for the intended purpose.

## 2. Results and Discussion

### 2.1. Development of AQbD Compliant Chaotropic Chromatography Method

The objective of this research was to efficiently develop a reliable and robust method for the analysis of ZPS and its five impurities using the AQbD approach, also known as robust optimization [18]. In developing analytical methods using the AQbD principles, the analytical target profile (ATP) must first be defined. The ATP is related to the validation criteria that must be met according to the ICH Q2 guideline [19], the critical method attributes (CMAs), or criteria for performance evaluation, and the critical method parameters (CMPs), or factors that influence the ATP (Table 1).

ZPS and its five impurities are substances with basic character. Based on our previous experience with such solutes [18,20,21], we decided to add column friendly chaotropic reagents to the mobile phase to achieve appropriate peak shape and retention. Anions, which are commonly part of the mobile phase in chaotropic chromatography, are hexafluorophosphate, perchlorate, or trifluoroacetate. Their presence in the mobile phase leads to the increase in retention, efficiency, and separation selectivity of examined fully protonated basic analytes due to different complex mechanisms that are thoroughly described by Vemić et al. [20]. Briefly, there are three different processes that support the effect of chaotropic ions on the retention of basic solutes: (i) protonated basic solutes and chaotropic anions can form ion pairs, which are retained at the stationary phase by a reversed-phase mechanism; (ii) disruption of the solute’s solvation shell by the chaotropic anions increases its hydrophobicity and, consequently, the retention; (iii) adsorption of the chaotropic anions on the stationary phase surface leading to the development of an electrostatic component in the general hydrophobic solute retention mechanism. The composition of the mobile phase, the chemistry of the stationary phase, and the particle size were varied in the initial phase of the method development due to the different lipophilicity of the analyzed substances. Wavelength was determined from available literature data [4,5]. The following columns were selected for the initial experiments: Zorbax Eclipse XDB C18 (150 × 4.6 mm, 5 µm), Zorbax Eclipse XDB C8 (150 × 4.6 mm, 5 µm), XTerra C18 (150 × 4.6 mm, 3.5 µm), XTerra C8 (150 × 4.6 mm, 3.5 µm), and XBridge C8 (100 × 3 mm, 3.5 µm). As the most important parameter in the development of the chaotropic chromatography method, the concentration of the chaotropic agent was first investigated in the preliminary experiments. The “chaotropic sensitivity” of a solute is proportional to its hydrophobicity, and the hexafulorophosphate anion typically leads to a very pronounced increase in the retention of more lipophilic solutes [20]. Considering the wide range of lipophilicity (*logD*) of the solutes studied (Figure 1), trifluoroacetate was not tested as the weakest chaotropic chromatography agent. Acceptable retention and peak shape of ZPS and its impurities was achieved with the perchlorate anion, while hexafluorophosphate was not tested because it could significantly increase the retention of the impurities III, IV, and V.

The mobile phase was a mixture of 50 mM or 100 mM aqueous solution of perchloric acid, pH 2.5, and acetonitrile or methanol. The tests were performed with isocratic and gradient elution of the mobile phase. Isocratic elution of the mobile phase did not result in adequate separation of the impurities III and V on the stationary phase, and the peak shape of the impurity II was not adequate. The preliminary results indicated that a gradient elution, with methanol as organic modifier, should be used for further development of the method, namely a linear gradient elution on XTerra C8 (150 × 4.6 mm, 3.5 µm) column. The perchlorate anion provided an appropriate starting position for further retention and separation adjustment on C8 stationary phase. The perchlorate concentration in the robust optimization was fixed at 100 mM once the appropriate peak shape and acceptable retention of the studied solutes were achieved. The pH of the aqueous phase only slightly affected the retention once complete protonation of the analytes under study was achieved. Therefore, the aqueous part of the mobile phase was a 100 mM aqueous solution of perchloric acid, pH 2.5.

To further fine-tune the baseline separation of ziprasidone and its five impurities, robust optimization of the selected CMPs was the next step. The initial methanol fraction, the final methanol fraction, and the duration of the gradient were selected as CMPs. The retention time of the impurity V peak (*t__imp V_*), the mean value of the peak widths according to the USP (*<W_USP_>*), and the time elapsed from the elution of the first to the elution of the last peak (*t__imp. V_ − t__imp. I_*) were monitored as critical responses of the system. During the preliminary experiments, in addition to the peaks of the studied impurities, another peak was observed on the chromatograms, the peak of an unknown impurity named impurity *x*. Due to its retention behavior, impurity *x* forms a critical pair with the peak of impurity III. Consequently, the retention time of the beginning of impurity III peak (*t_b_imp. III_*) and the retention time of the end of impurity *x* peak (*t_e_imp. x_*) were also monitored. The additional CMA *S* = *t_b_imp. III_ − t_e_imp. x_* was indirectly modelled, i.e., calculated from the corresponding retention times of the end of first eluting peak and retention time of the beginning of second eluting peak [22].

Plan of experiments was defined by the Box–Behnken design (Table 2). A total of 16 experiments were performed, of which 4 experiments were performed at the central point to estimate the experimental error. The experiments were performed randomly to reduce the influence of external factors. The obtained response values are shown in Table 2.

Based on the experimental results, mathematical models describing the dependence of the critical responses of the system on the studied factors were created. The dependence of *t__imp. V_*, *t__imp. V_* − *t__imp. I_*, *t_e_imp. x_* and *t_b_imp. III_* on the investigated factors are described by quadratic mathematical models, while, for *<W_USP_>*, a linear mathematical model was created. The adequacy of the formed mathematical models was confirmed using the analysis of variances *(ANOVA*) test. High values of determination coefficients (*R^2^*, *adjusted R^2^*, and *predicted R^2^*) and a statistically insignificant lack of fit value were obtained (Table 3). In this way, it was confirmed that the defined models can successfully explain the influences of the studied factors and factor interactions, and that the responses of the system can be successfully predicted for any combination of CMPs within the limits of the experimental space.

In the next step, the statistical significance of the influence of the examined factors and factor interactions on the critical responses of the system was examined. Statistical significance was estimated based on the *p*-value, i.e., Student’s *t* test. Although all examined factors show a statistically significant influence on the system responses, based on the absolute values of the coefficients of the mathematical models, it was concluded that the *x*_3_ had the greatest influence on the observed responses. The significantly higher absolute value of the coefficients of the *x*_2_ factor compared to the value of the coefficients of the *x*_1_ factor indicated that the response values are more sensitive to the change in the final methanol content than to the change in the initial methanol content. Based on the positive sign of the coefficient of the factor *x*_3_, it can be concluded that, with the extension of the duration of the gradient, the values of the system’s critical responses will increase. Based on the negative sign of the coefficients of factors *x*_1_ and *x*_2_, it can be concluded that an increase in the initial and final fraction of methanol will lead to a decrease in the values of the monitored responses of the system.

### 2.2. Computation of 3D-DS and 2D-DS for the Selection of Working Point

**Figure 2:**
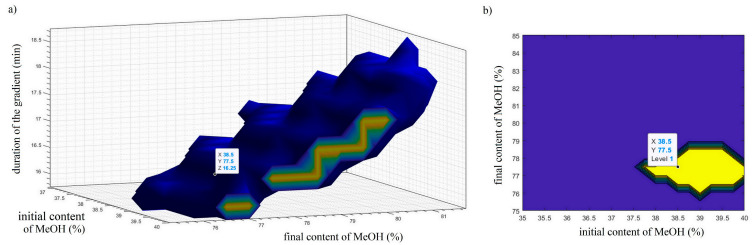
(**a**) Three-dimensional representation of DS for the predefined CMAs achieved with probability π ≥ 80%. (**b**) Two-dimensional representation of DS after setting a fixed value for gradient duration 16.25 min. The yellow part corresponds to the region of the design space where the working point should be situated.

After describing and studying the retention behavior of the analyzed analytes based on the formed mathematical models, the design space (DS) was computed using the following CMAs criteria: *t__imp. V_* < 15.5 min, *t__imp. V_ − t__imp. I_* < 12 min, *S* ≥ 0 min, and *<W_USP_>* ≤ 0.235 min. The experimental space was divided by discretization of the factors: initial methanol fraction [35:0.25:40], final methanol fraction [75:0.5:85], and gradient duration [15:0.5:20]. Therefore, the experimental domain was divided into 21 levels for *x*_1_ × 21 levels, for *x*_2_ × 11 levels, and for *x*_3_ = 4851 points to be analyzed. As DS is an area in which the CMAs meet the set criteria with an acceptable degree of probability (according to ATP π = 80%), Monte Carlo simulations were applied to achieve the defined quality. In each of the 4851 points of the experimental space, 5000 iterations were applied. In order to obtain distribution of *t__imp. V_*, *t__imp. V_ − t__imp. I_*, and <*W_USP_*>, the uniform error distribution, equal to the calculated standard error, was added to the estimate of each model coefficient. Through the indirect modeling, the distribution of *S* for the given chromatographic conditions was also obtained. The DS computed in this way is illustrated in Figure 2.

**Figure 3:**
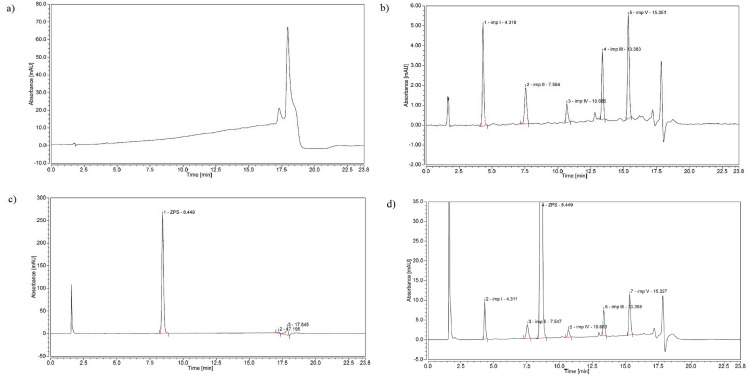
Chromatograms obtained under conditions suggested by the working point: (**a**) placebo mixture of excipients, (**b**) laboratory mixture of impurities at LOQ, (**c**) ziprasidone capsule’s solution containing 50 μg mL^−1^ of ZPS, and (**d**) laboratory mixture containing 500 μg mL^−1^ of ZPS and impurities at specification level (0.2%).

Since the robustness of the defined qualitative performance of the method (the selected CMAs) is guaranteed within the formed DS, any point in it can be chosen as a working point. The working point chosen in this work is characterized by the following conditions: initial methanol content 38.5%, final methanol content 77.5%, and gradient duration 16.25 min. At the working point, a verification experiment was performed, in which the solution of placebo, laboratory mixture of the tested impurities, and the ziprasidone capsule’s solution were analyzed under selected chromatographic conditions (Figure 3).

Based on the obtained values of *t__nec. V_* = 15.351 min, *S* = 0.02 min, *<W_USP_>* = 0.233 min, and *t__imp. V_ − t__imp. I_* = 11.033 min, it was confirmed that, under the chromatographic conditions at the selected working point, the method met the previously set CMA criteria (Table 1) confirming the quality of the developed method.

### 2.3. Quantitative Robustness Testing

AQbD-compliant analytical methods focus on separation or resolution and retention, i.e., qualitative CMAs [23]. Therefore, the robustness of quantitative performances (i.e., peak area of investigated substances) should be tested as a part of method validation. The significant/influencing factors, causing variability in the quantitative performance of the developed method, were identified using the Plackett–Burman design. According to the findings from the method development and robust optimization experiments, seven quantitative factors and four dummy factors were included in the experimental design (Table 4).

The factors related to the HPLC system, such as flow rate and detection wavelength, were not investigated in the AQbD compliant method development but in the quantitative robustness tests, because they could have a significant impact on the quantitative performances of the method. The peak areas of ZPS and its five impurities were selected as quantitative responses (*P__API_*, *P__imp I_*, *P__imp II_*, *P__imp III_*, *P__imp IV_*, *P__imp V_*). The plan of experiments and experimentally obtained responses are shown in Table 4. The resulting main effects models had acceptable statistical parameters, and the estimates for dummy factor effects were not statistically significant, indicating that no inestimable interactions were leaking into the estimable effects [18]. Only two quantitative responses, *P__imp II_* and *P__imp V_*, were found to be significantly affected, the first by mobile phase flow rate (*N*) and the second by gradient duration (*D*). Since the developed method is intended for the control of impurities, it is worth mentioning that the mobile phase flow rate (*N*) and the gradient duration (*D*) should be strictly controlled. This can be efficiently achieved by regular qualification and performance maintenance of the HPLC system.

### 2.4. Method Validation

Finally, the suitability of the developed method for the defined ATP was confirmed by testing the sensitivity, selectivity, linearity, accuracy, and precision. Sensitivity was based on the experimental determination of LOD and LOQ for the impurities by a signal-to-noise (S/N) approach, i.e., by continuous dilution of reference standard solutions until the obtained response (signal) to noise ratio were S/N of 3:1 and 10:1 for the estimation of LOD and LOQ, respectively (Table 5). The calculated regression parameters and recovery values (Table 5) met the acceptance criteria for linearity and accuracy, respectively [24]. The precision of the method was evaluated by calculating the RSD: ZPS (0.40%), impurity I (3.85%), impurity II (5.80%), impurity III (3.17%), impurity IV (6.25%), and impurity V (8.10%), and the values obtained met the acceptance criteria (RSD 2% for active ingredients, RSD 15% for impurities with the specification limit of 0.1% to 0.5%) [24]. The content of ZPS in the analyzed tablets was 95.00%, while the impurities were below the LOQ.

## 3. Materials and Methods

### 3.1. Reagents and Chemicals

Reference standard of ZPS hydrochloride and its impurities I, II, III, IV, and V were kindly donated by Krka, Novo Mesto, Slovenia. Organic solvents, which were used for the preparation of mobile phase and solvent, HPLC gradient grade methanol, and HPLC gradient grade acetonitrile, were purchased from Fisher Chemical, Fisher Scientific (Hampton, NH, USA). Water was purified to HPLC grade with water purification system Adrona Onsite^+^ Bio, Adrona Ltd., Latvia. Additive to aqueous phase, perchloric acid was purchased from Sigma-Aldrich, Merck KGaA, Darmstadt, Germany. Sodium hydroxide solution (10 M), which was used to adjust the pH value of the aqueous phase, was purchased from Fisher Chemical, Fisher Scientific (Hampton, NH, USA). Hydrochloric acid (37%) was obtained from Sigma-Aldrich, Merck KGaA, Darmstadt, Germany. All used chemicals were of the analytical grade. Pharmaceutical dosage form, ZPS capsules, used in validation studies, were kindly donated by the supreme regulatory body of Serbia—Medicines and Medical Devices Agency of Serbia.

### 3.2. Chromatographic Conditions

The experiments were performed on Vanquish Core HPLC system (Thermo Fisher Scientific, Germering, Germany) equipped with quaternary pump, autosampler, degasser, photodiode array detector (PDA), and with software Chromeleon™ Chromatography Data System (CDS) 7.3 for data acquisition. The initial experiments were performed on five columns: Zorbax Eclipse XDB C18, 150 × 4.6 mm, 5 µm (Agilent Technologies, Santa Clara, CA, USA), Zorbax Eclipse XDB C8, 150 × 4.6 mm, 5 µm (Agilent Technologies, Santa Clara, CA, USA), XTerra C18, 150 × 4.6 mm, 3.5 µm (Waters Corporation, Milford, MA, USA), XTerra C8, 150 × 4.6 mm, 3.5 µm (Waters Corporation, Milford, MA, USA), and XBridge C8, 100 × 3 mm, 3.5 µm (Waters Corporation, Milford, MA, USA). The column selected for optimization and validation studies was XTerra C8 (150 × 4.6 mm, 3.5 µm).

The programs of gradient (initial methanol content, final methanol content, and gradient duration) were changed according to the experimental plan defined by Box–Behnken design (Table 2).

The aqueous part of mobile phase was prepared by adding an appropriate amount of perchloric acid in HPLC grade water, to obtain the required molarity of final of the solution, and by adding sodium hydroxide 10 M solution to adjust the desired pH (2.5). All prepared mobile phases for method development and validation phase were filtered through a 0.45 µm nylon filter membrane (Agilent Technologies, Santa Clara, CA, USA) and degassed under vacuum prior to use.

Other chromatographic conditions were kept constant: detection wavelength was 230 nm, mobile phase flow rate 1 mL min^–1^, and column and autosampler temperature were set on 30 °C and 15 °C, respectively. The injection volume was fixed at 15 μL.

### 3.3. Standard Solutions for Method Development and Robustness Testing

Stock solutions were prepared by dissolving the appropriate amounts of reference standard substances in the solvent mixture acetonitrile–methanol–water–hydrochloric acid (12:48:40:0.04%, *v*/*v*) to obtain the concentrations of 1 mg mL^–1^ for ZPS hydrochloride and 100 μg mL^–1^ for impurity I, II, III, IV, and V. Stock solutions were diluted in the solvent mixture methanol–water (30:70%, *v*/*v*) to obtain a working solution of ZPS hydrochloride with a concentration of 100.0 µg mL^–1^ and working solutions of impurities with concentrations of 10.0 µg mL^–1^.

### 3.4. Standard Solutions for Method Validation

#### 3.4.1. Solutions for Selectivity Estimation

A mixture of excipients (lactose monohydrate, pregelatinized maize starch, magnesium stearate) and placebo was prepared in the concentration ratio corresponding to the content in capsules. It was treated in the same manner as the sample used for the precision estimation. A sample solution containing 500 µg mL^−1^ of ZPS and a standard solution containing the impurities at the concentrations corresponding to their limits of quantification were used to prove the method selectivity.

#### 3.4.2. Solutions for Linearity Estimation

Linearity for ZPS hydrochloride was studied by preparing five standard solutions in a concentration range of 25.0–75.0 µg mL^–1^. The solutions were prepared by diluting ZPS hydrochloride stock solution (200 μg mL^–1^) with the methanol–water mixture (30:70%, *v*/*v*). Five standard solutions for each impurity (I, II, III, IV, and V) were prepared in a concentration range of 0.50–1.20 µg mL^–1^ by diluting appropriate stock solution (100 μg mL^–1^) with the methanol–water mixture (30:70%, *v*/*v*).

#### 3.4.3. Solutions for Accuracy Estimation

Accuracy was tested in triplicate on three concentration levels, 80%, 100%, and 120%, for ZPS hydrochloride and LOQ, and 100% and 120% for each impurity. Stock solutions for accuracy estimation were prepared by dissolving a mixture of placebo and ZPS hydrochloride reference standard, as well as a mixture of placebo and reference standard of each impurity in acetonitrile–methanol–water–hydrochloric acid solvent mixture (12:48:40:0.04%, *v*/*v*) in an ultrasonic bath for 15 min. After filtration through a syringe nylon membrane filter (0.45 μm), stock solutions were diluted in the solvent mixture methanol–water (30:70%, *v*/*v*) to obtain working solutions containing 40.0, 50.0, and 60.0 μg mL^–1^ of ZPS hydrochloride and 0.50, 1.00, and 1.20 μg mL^–1^ of each impurity—I, II, III, IV, and V.

#### 3.4.4. Solutions for Precision Estimation

Stock solutions for estimation of the precision of the method for the quantification of ZPS hydrochloride (active substance) and its impurities were prepared by separately weighing the content of capsules corresponding to 10 mg, i.e., 250 mg of ZPS hydrochloride. Weighed capsule contents were extracted with the solvent mixture acetonitrile–methanol–water–hydrochloric acid (12:48:40:0.04%, *v*/*v*) in 50 mL volumetric flasks using an ultrasonic bath for 15 min. Each of the volumetric flasks were filled with the same solvent mixture to obtain concentrations of 200 μg mL^–1^ and 5 mg mL^−1^ of ZPS hydrochloride. After the filtration stock solutions were diluted in the solvent mixture methanol–water (30:70%, *v*/*v*), we obtained six solutions containing 50.0 μg mL^–1^ ZPS hydrochloride, for the estimation of the precision of the active substance determination, and 500 μg mL^–1^ of ZPS hydrochloride, for the estimation of the precision of the determination of impurities.

### 3.5. Software

Calculations of *pKa*, *logD*, and *logP* values and drawings of structures for tested analytes were performed using ChemAxon’s Chemicalize platform (ChemAxon Ltd., Budapest, Hungary). The experimental plan for method optimization and the Placket–Burman experimental plan for assessing the quantitative robustness of the method were defined, and the corresponding data analysis was performed using the Design-Expert^®^ 11.0.0 (Stat-Ease Inc., Minneapolis, MN, USA). MATLAB^®^ R2018b (The MathWorks, Inc., Natick, MA, USA) was used for Monte Carlo simulations and graphical presentation of DS for method development by the AQbD approach. For additional calculations, Microsoft Excel 2010 (Microsoft, Redmond, WA, USA) was used.

## 4. Conclusions

A reliable, robust, and AQbD-compliant chaotropic chromatography method for efficient baseline separation and accurate determination of ziprasidone and its five impurities from capsules was developed. The DS was defined based on a probability π ≥ 80% for the selected CMAs within the predefined acceptance values (t__imp. V_ < 15.5 min, *t__imp. V_ − _t_imp. I_ <* 12 min, *S* ≥ 0 min, and *<W_USP_>* ≤ 0.235 min). A working point was selected from the middle of the defined DS that provided good agreement between experimentally determined and predicted CMAs. Application of DoE methodology and risk assessment using Monte Carlo simulations ensured the robustness of the qualitative performances of the method within the bounds of DS. The quantitative performance of the developed method was also evaluated by robustness tests to investigate the effects of selected chromatographic factors on the quantitative responses (*P__API_*, *P__imp I_*, *P__imp II_*, *P__imp III_*, *P__imp IV_*, *P__imp V_*). It was found that only *P__imp II_* and *P__imp V_* can be significantly affected by mobile phase flow rate (*N*) and by gradient duration (*D*), respectively. Finally, the reliability and applicability of the developed method were confirmed using the appropriate validation experiments. It was shown that all the criteria defined by ATP can be achieved in less than 16 min.

## Figures and Tables

**Table 1:** Key elements for the AQbD concept applied on chaotropic chromatography method development for ziprasidone and its five impurities assay in capsules.

	Gradient Elution Mode
**ATP**	The efficient baseline separation and accurate determination of ziprasidone and its five impurities assay in capsules. *Probability*: π ≥ 80% *Separation factors*: *S* ≥ 0 min *Retention time of the last eluting peak*: *t__imp. V_* < 15.5 min *Mean value of peak widths according to the USP: <W_USP_>* ≤ 0.235 min *The time elapsed from the elution of the first to the elution of the last peak: t__imp. V_ − t__imp. I_* < 12 min *Recovery values*: 98.0–102.0% for ziprasidone, 70.0–130.0% for impurities. *Limit of detection*: not less than 0.05%
**CMP**	*x*_1_—initial content of methanol in the mobile phase (%, *v*/*v*) *x*_2_—the final content of methanol in the mobile phase (%, *v*/*v*) *x*_3_—the duration of the gradient (min)
**CMA**	*S* (min) *t__imp. V_* (min) *<W_USP_>* (min) *t__imp. V_ − t__imp. I_* (min)

ATP—analytical target profile; CMP—critical method parameters; CMA—critical method attributes; *S*—separation factor for unknown impurity *x* an impurity III (*S* = *t_b_imp III_ − t_e_imp x_*); *t_e_imp. x_*—the retention time of the end of the unknown impurity peak; *tb_imp. III*—the retention time of the beginning of the impurity III peak; *t__imp. V_*—the retention time of the impurity V peak; *<W_USP_>*—mean value of peak widths according to the USP; *t__imp. V_ − t__imp. I_*—the time elapsed from the elution of the first to the elution of the last peak.

**Table 2:** Experiment plan and experimentally obtained response values.

№	*x*_1_	*x*_2_	*x*_3_	*t_e_imp. x_*	*t_b_imp. III_*	*t__imp. V_*	*<W_USP_>*	*t__imp. V_ − t__imp. I_*
**1**	35	75	17.5	15.00	15.03	17.21	0.24	12.405
**2**	40	75	17.5	13.66	13.73	16.14	0.25	12.302
**3**	35	85	17.5	13.11	13.11	15.00	0.21	10.248
**4**	40	85	17.5	12.04	12.04	14.04	0.22	10.192
**5**	35	80	15.0	12.81	12.81	14.62	0.21	9.860
**6**	40	80	15.0	11.84	11.84	13.77	0.22	9.915
**7**	35	80	20.0	15.24	15.24	17.43	0.24	12.589
**8**	40	80	20.0	13.84	13.84	16.23	0.25	12.358
**9**	37.5	75	15.0	13.24	13.24	15.31	0.23	11.012
**10**	37.5	85	15.0	11.63	11.63	13.35	0.20	9.063
**11**	37.5	75	20.0	15.62	15.63	18.14	0.26	13.832
**12**	37.5	85	20.0	13.72	13.73	15.82	0.23	11.515
**13**	37.5	80	17.5	13.38	13.42	15.53	0.23	11.257
**14**	37.5	80	17.5	13.39	13.40	15.52	0.23	11.242
**15**	37.5	80	17.5	13.50	13.51	15.59	0.23	11.298
**16**	37.5	80	17.5	13.50	13.50	15.58	0.23	11.289

*x*_1_—initial content of methanol in the mobile phase (%, *v*/*v*); *x*_2_—the final content of methanol in the mobile phase (%, *v*/*v*); *x*_3_—the duration of the gradient (min); *t_e_imp. x_*—the retention time of the end of the unknown impurity peak (min); *t_b_imp. III_*—the retention time of the beginning of the impurity III peak (min); *t__imp. V_*—the retention time of the impurity V peak (min); *<W_USP_>*—mean value of peak widths according to the USP; *t__imp. V_ − t__imp. I_*—the time elapsed from the elution of the first to the elution of the last peak (min).

**Table 3:** Model coefficients calculated for coded factor values and statistically significant parameters obtained by *ANOVA*.

	*t__imp. V_*	*t_e_imp x_*	*t_b_imp III_*	*<W_USP_>*	*t__imp V_ − t__imp I_*
***b*_0_**	15.554 *	13.443 *	13.458 *	0.2289 *	11.272 *
***b*_1_**	−0.511 *	−0.598 *	−0.593 *	0.0041 *	−0.042 *
***b*_2_**	−1.076 *	−0.878 *	−0.890 *	−0.0132 *	−1.067 *
***b*_3_**	1.321 *	1.113 *	1.115 *	0.0152 *	1.306 *
***b*_12_**	0.029	0.068	0.057 *	/	0.012
***b*_13_**	−0.087 *	−0.108 *	−0.108 *	/	−0.072 *
***b*_23_**	−0.090 *	−0.073 *	−0.073 *	/	−0.092 *
***B*_11_**	−0.050 *	−0.055	−0.053 *	/	−0.080 *
***b*_22_**	0.094 *	0.065 *	0.072 *	/	0.095 *
***b*_33_**	0.006	0.045	0.028	/	−0.011
***R* ^2^**	0.9998	0.9993	0.9994	0.9838	0.9999
***adj. R* ^2^**	0.9996	0.9982	0.9986	0.9797	0.9996
***pred. R* ^2^**	0.9995	0.9984	0.9977	0.9711	0.9989

*b*_0_—constant; *b*_1_, *b*_2_, *b*_3_—coefficients of main factors; *b*_11_, *b*_22_, *b*_33_—quadratic coefficients; *b*_12_, *b*_13_, *b*_23_—interaction coefficients; *R^2^*—coefficient of determination; *adj. R^2^*—adjusted coefficient of determination; *pred. R^2^*—prediction coefficient of determination; *t_e_imp. x_*—the retention time of the end of the unknown impurity peak; *t_b_imp. III_*—the retention time of the beginning of the impurity III peak; *t__imp. V_*—the retention time of the impurity V peak; *<W_USP_>*—mean value of peak widths according to the USP; *t__imp. V_ − t__imp. I_*—the time elapsed from the elution of the first to the elution of the last peak. * statistically significant values of coefficients whose *p* < 0.05.

**Table 4:** Plan of experiments defined by Plackett–Burman design and experimentally obtained results.

№	*A*	*B*	*C*	*D*	*E*	*F*	*G*	*H*	*J*	*K*	*L*	*P__imp I_*	*P__imp II_*	*P__API_*	*P__imp III_*	*P__imp IV_*	*P__imp V_*
**1**	39.5	1	76.5	16.5	1	10	−1	0.9	−1	35	229	1417.35	353.84	9069.79	418.65	281.58	1378.96
**2**	37.5	1	78.5	16	1	10	1	0.9	−1	25	231	1640.374	369.832	10,040.28	405.841	238.698	1451.263
**3**	39.5	−1	78.5	16.5	−1	10	1	1.1	−1	25	229	1095.628	344.924	7130.304	254.497	212.253	1177.324
**4**	37.5	1	76.5	16.5	1	5	1	1.1	1	25	229	1051.206	264.087	6899.781	254.641	224.772	1053.161
**5**	37.5	−1	78.5	16	1	10	−1	1.1	1	35	229	1640.114	243.709	10624.03	408.023	283.986	1522.191
**6**	37.5	−1	76.5	16.5	−1	10	1	0.9	1	35	231	1301.494	361.236	8404.344	246.173	258.071	1176.329
**7**	39.5	−1	76.5	16	1	5	1	1.1	−1	35	231	1376.018	215.475	9289.761	203.823	221.739	1327.625
**8**	39.5	1	76.5	16	−1	10	−1	1.1	1	25	231	1084.411	325.384	6888.604	258.061	195.279	1093.397
**9**	39.5	1	78.5	16	−1	5	1	0.9	1	35	229	1768.829	306.912	10,205.74	531.56	297.964	1532.409
**10**	37.5	1	78.5	16.5	−1	5	−1	1.1	−1	35	231	2001.457	289.047	9661.038	371.323	272.7	1340.322
**11**	39.5	−1	78.5	16.5	1	5	−1	0.9	1	25	231	1202.518	270.312	7721.945	269.349	229.951	1201.462
**12**	37.5	−1	76.5	16	−1	5	−1	0.9	−1	25	229	1625.379	400.925	10207.9	254.525	320.083	1531.502

*A*—initial content of methanol in the mobile phase (%, *v*/*v*); *B*—dummy 1; *C*—the final content of methanol in the mobile phase (%, *v*/*v*); *D*—the duration of the gradient (min); *E*—dummy 2; *F*—duration of re-equilibration (min); *G*—dummy 3; *H*—mobile phase flow rate (mL min^−1^); *J*—dummy 4; *K*—column temperature (°C); *L*—detection wavelength (nm); *P__imp I_*—peak area of impurity I; *P__imp_*_*II*_—peak area of impurity II; *P__API_*—ziprasidone peak area; *P__imp III_*—peak area of impurity III; *P__imp IV_*—peak area of impurity IV; *P__imp V_*—peak area of impurity V.

**Table 5:** Validation parameters of the proposed LC method.

Substance	LOD (μg mL^−1^)	Linearity					
		Concentration Range (µg mL^–1^)	*a*	*b*	*r*	Concentration Level (µg mL^–1^)	Recovery ** (%)
Ziprasidone	-	25–75	0.7832	−0.032	0.9999	40	98.65 (0.76%) ***
						50	98.56 (0.88%)
						60	98.57 (0.34%)
Impurity I	0.15	0.5 *–1.2	1.0216	0.0945	0.9989	0.5	97.01 (0.61%) ***
						1.0	102.80 (0.24%)
						1.2	105.90 (0.16%)
Impurity II	0.15	0.5 *–1.2	0.6459	0.0167	0.9965	0.5	105.37 (0.78%) ***
						1.0	93.77 (3.99%)
						1.2	95.77 (0.13%)
Impurity III	0.15	0.5 *–1.2	0.82092	0.0675	0.9989	0.5	97.82 (0.66%) ***
						1.0	99.36 (0.39%)
						1.2	104.65 (1.24%)
Impurity IV	0.15	0.5 *–1.2	0.3345	−0.0313	0.9958	0.5	107.57 (3.26%) ***
						1.0	98.42 (2.90%)
						1.2	106.35 (8.15%)
Impurity V	0.15	0.5 *–1.2	1.5028	0.0173	0.9949	0.5	101.95 (1.06%) ***
						1.0	85.69 (1.07%)
						1.2	89.02 (0.31%)

*a*—slope, *b*—intercept, *r*—correlation coefficient (acceptance value > 0.99 for active ingredients, >0.98 for related compounds). * concentration corresponding to LOQ level. ** Recovery: acceptance value 98.0–102.0% for active ingredients, 70.0–130.0% for impurities with the specification limit from 0.1% to 0.5%. *** RSD of triplicated determination at 80%, 100%, and 120% for ZPS hydrochloride and LOQ, 100% and 120% for each impurity (RSD 2% for active ingredients, RSD 15% for impurities with the specification limit of 0.1% to 0.5%).

## Data Availability

Data is contained within the article.
